# Earnings of Employed and Self-employed US Health Care Professionals, 2001 to 2015

**DOI:** 10.1001/jamanetworkopen.2018.0431

**Published:** 2018-06-29

**Authors:** Kamyar Nasseh, Marko Vujicic

**Affiliations:** 1 Health Policy Institute, American Dental Association, Chicago, Illinois

## Abstract

**Importance:**

Over the last 15 years, the health care practitioner landscape has changed significantly. Fewer practitioners are self-employed and more are employed by for-profit or nonprofit organizations. These shifts can have an impact on annual labor earnings.

**Objectives:**

To examine trends in self-employment and employment and to assess the gap in annual labor earnings between self-employed and employed US health care professionals from 2001 to 2015.

**Design, Setting, and Participants:**

Survey study in which data on employment type (self-employed, employed by private sector, or employed by government) and annual labor earnings for 50 states and the District of Columbia were extracted from the 2001 to 2015 American Community Survey. The analyses were restricted to 175 714 self-identified dentists, physicians, pharmacists, optometrists, podiatrists, chiropractors, and physical therapists aged 30 years and older who worked at least 40 weeks per year and 20 hours per week. Controlling for age, sex, race/ethnicity, year, and state of residence, median regression models were used to measure the gap in annual labor earnings between self-employed and employed health care professionals.

**Main Outcomes and Measures:**

Annual labor earnings, defined as the sum of self-employment and wages or salary income.

**Results:**

Our sample of 175 714 respondents included 99 077 physicians, 20 008 dentists, 26 143 pharmacists, 4238 optometrists, 6076 chiropractors, 1164 podiatrists, and 19 008 physical therapists. The weighted percentage of self-employed physicians decreased from 35.2% (95% CI, 34.4%-36.1%; 6807 of 18 726 physicians) in 2001 through 2005 to 24.7% (95% CI, 24.2%-25.2%; 10 974 of 41 205 physicians) in 2011 through 2015. The percentage of self-employed dentists decreased from 73.0% (95% CI, 71.2%-74.8%; 3117 of 4153 dentists) in 2001 through 2005 to 65.1% (95% CI, 63.7%-66.4%; 5260 of 7820 dentists) in 2011 through 2015. Among physicians, the regression-adjusted earnings gap reversed from $19 679 (95% CI, $14 431-$24 927; *P* < .001) during 2001 through 2005 to −$10 623 (95% CI, −$14 547 to −$6699; *P* < .001) during 2011 through 2015. Among dentists, the regression-adjusted earnings gap narrowed from $30 448 (95% CI, $23 040-$37 855; *P* < .001) during 2001 through 2005 to $21 291 (95% CI, $15 723-$26 859; *P* < .001) during 2011 through 2015. From 2001 to 2015 the earnings gap also reversed among pharmacists, optometrists, and podiatrists. The regression-adjusted earnings gap narrowed among chiropractors and physical therapists.

**Conclusions and Relevance:**

Since 2001, the percentage of health care professionals who are self-employed declined, and the gap in earnings between self-employed and employed health care professionals narrowed.

## Key Points

**Question:**

Since 2001, what changes have occurred in the gap in annual earnings between health care professionals who are self-employed and those who are employed by for-profit or nonprofit organizations?

**Findings:**

In this survey study examining responses from self-identified dentists, physicians, pharmacists, chiropractors, optometrists, podiatrists, and physical therapists participating in the American Community Survey between 2001 and 2015, the percentage identifying themselves as self-employed decreased. The gap in earnings between these professionals and those employed by organizations either narrowed or reversed.

**Meaning:**

These trends may represent a move of health care professionals toward larger provider groups. Additional research is warranted to determine the driving forces behind the shift away from self-employment and the shrinking earnings gap between health care professionals who are self-employed and those employed by for-profit or nonprofit organizations.

## Teaser

This study uses responses from health care professionals who participated in the American Community Survey between 2001 and 2015 to identify trends in self-employment and changes in the earnings differences between self-employed professionals and those employed by for-profit or nonprofit organizations.

## Introduction

In health care, there has been a shift away from self-employment.^1^ Little is known about the gap in earnings between self-employed health care professionals and those employed by for-profit or nonprofit organizations (referred to in this article as *employed*). We analyzed this gap for dentists, physicians, pharmacists, optometrists, podiatrists, chiropractors, and physical therapists. These professions have a mix of self-employment and employment. We did not examine nurses or physician assistants because they are predominantly employed by physician practices or hospitals.^2^

## Methods

We used data from the nationally representative American Community Survey (ACS) collected between 2001 and 2015.^3^ The response rate for this survey was more than 90%.^4^ This study followed the Strengthening the Reporting of Observational Studies in Epidemiology (STROBE) reporting guidelines. Institutional review board approval was not required because the study used publicly available data.

Our analysis was limited to respondents aged 30 years and older who worked at least 40 weeks in the previous year and at least 20 hours per week and had positive annual labor earnings. We defined earnings as the sum of self-employment and wages or salary income. Using the all-items Consumer Price Index, we inflated earnings to 2015 dollars. Employed health care professionals were those who self-reported that they were employed in private for-profit or nonprofit organizations. Self-employed professionals were those who self-reported that they were self-employed in an incorporated or unincorporated business or professional practice. We grouped federal, state, and local government employees together in a separate category. Survey respondents could choose only 1 response to the employment type question in the ACS.

We examined trends in employment type and median annual labor earnings in 5-year increments (2001-2005, 2006-2010, and 2011-2015). The ACS top-codes reported income at the 99.5 percentile for each state.^5^ To mitigate possible bias from top-coding, following Seabury et al,^6^ we estimated a median regression model to estimate the earnings gap (self-employed minus employed earnings) by profession, adjusted for age, sex, race/ethnicity, year, and state of residence. We used a significance threshold of .05 using a 2-sided test.

## Results

Our sample of 175 714 respondents included 99 077 physicians, 20 008 dentists, 26 143 pharmacists, 4238 optometrists, 6076 chiropractors, 1164 podiatrists, and 19 008 physical therapists. The weighted percentage of self-employed physicians decreased from 35.2% (95% CI, 34.4%-36.1%; 6807 of 18 726 physicians) in 2001 through 2005 to 24.7% (95% CI, 24.2%-25.2%; 10 974 of 41 205 physicians) in 2011 through 2015. The percentage of self-employed dentists decreased from 73.0% (95% CI, 71.2%-74.8%; 3117 of 4153 dentists) in 2001 through 2005 to 65.1% (95% CI, 63.7%-66.4%; 5260 of 7820 dentists) in 2011 through 2015. The percentage of self-employed professionals decreased among podiatrists, chiropractors, and optometrists from 2001 to 2015. Most physical therapists and pharmacists were employed throughout the study period (Table 1).

**Table 1.** Trends in Employment Type by Profession, 2001 to 2015

Type of Professional	Employment Type, % (95% CI)		
	2001-2005	2006-2010	2011-2015
Dentists (n = 20 008)			
Employed	22.6 (20.9-24.3)	25.0 (23.8-26.1)	30.4 (29.2-31.7)
Self-employed	73.0 (71.2-74.8)	70.7 (69.6-71.9)	65.1 (63.7-66.4)
Government	4.4 (3.6-5.1)	4.3 (3.8-4.8)	4.5 (3.9-5.1)
Physicians (n = 99 077)			
Employed	52.4 (51.5-53.3)	58.4 (57.9-59.0)	64.2 (63.6-64.8)
Self-employed	35.2 (34.4-36.1)	29.9 (29.4-30.4)	24.7 (24.2-25.2)
Government	12.4 (11.7-13.0)	11.7 (11.3-12.1)	11.1 (10.7-11.5)
Pharmacists (n = 26 143)			
Employed	83.4 (82.1-84.7)	86.3 (85.6-87.1)	87.2 (86.4-87.9)
Self-employed	8.2 (7.3-9.2)	6.5 (6.0-7.0)	5.3 (4.8-5.8)
Government	8.4 (7.4-9.4)	7.2 (6.6-7.8)	7.5 (6.9-8.1)
Optometrists (n = 4238)			
Employed	31.8 (27.7-35.8)	35.0 (32.4-37.6)	42.7 (39.8-45.6)
Self-employed	65.1 (60.9-69.2)	62.1 (59.5-64.7)	53.1 (50.2-56.0)
Government	3.2 (1.7-4.6)	2.9 (2.1-3.8)	4.2 (2.9-5.4)
Podiatrists (n = 1164)			
Employed	25.5 (19.4-31.5)	27.2 (22.3-32.2)	38.6 (32.5-44.8)
Self-employed	72.1 (66.0-78.3)	69.1 (64.1-74.2)	57.7 (51.5-63.9)
Government	2.4 (0.6-4.2)	3.6 (1.7-5.5)	3.6 (1.3-6.0)
Chiropractors (n = 6076)			
Employed	19.1 (16.3-21.9)	20.3 (18.3-22.2)	24.4 (22.1-26.7)
Self-employed	80.9 (78.1-83.7)	79.5 (77.6-81.5)	75.0 (72.7-77.3)
Government	NA^a^	0.2 (0.0-0.3)	0.6 (0.2-0.9)
Physical therapists (n = 19 008)			
Employed	79.5 (77.7-81.2)	78.9 (77.9-80.0)	82.2 (81.2-83.2)
Self-employed	11.8 (10.5-13.2)	12.9 (12.0-13.7)	10.9 (10.1-11.8)
Government	8.7 (7.4-9.9)	8.2 (7.5-8.9)	6.8 (6.2-7.5)

Abbreviation: NA, not available.

^a^ No survey results were available for this category.

Among self-employed physicians, median (interquartile range [IQR]) annual earnings increased from 2001 through 2005 ($223 805 ([$135 639-$376 413]) to 2011 through 2015 ($249 767 [$111 058-$365 863]). Median (IQR) annual earnings among employed physicians increased from 2001 through 2005 ($179 350 [$92 767-$302 850]) to 2011 through 2015 ($200 253 [$104 287-$363 914]) (Table 2). Among physicians, the regression-adjusted earnings gap reversed from $19 679 (95% CI, $14 431-$24 927; *P* < .001) during 2001 through 2005 to −$10 623 (95% CI, −$14 547 to −$6699; *P* < .001) during 2011 through 2015. Median (IQR) annual earnings among self-employed dentists declined from $188 539 ($104 442-$265 932) in 2001 through 2005 to $158 841 ($95 011-$312 860) in 2011 through 2015. Among employed dentists, median (IQR) annual earnings held steady from 2001 through 2005 ($133 008 [$93 046-$217 023]) to 2011 through 2015 ($131 166 [$85 818-$201 924]). The regression-adjusted earnings gap narrowed from $30 448 (95% CI, $23 040-$37 855; *P* < .001) during 2001 through 2005 to $21 291 (95% CI, $15 723-$26 859; *P* < .001) during 2011 through 2015. From 2001 to 2015 the earnings gap reversed among pharmacists, optometrists, and podiatrists. The regression-adjusted earnings gap narrowed among chiropractors and physical therapists (Figure).

**Table 2.** Trends in Unadjusted Annual Median Earnings by Profession and Employment Type, 2001 to 2015

Type of Professional	Unadjusted Annual Earnings, Median (IQR), $^a^		
	2001-2005	2006-2010	2011-2015
Dentists (n = 20 008)			
Employed	133 008 (93 046-217 023)	134 532 (89 688-218 614)	131 166 (85 818-201 924)
Self-employed	188 539 (104 442-265 932)	168 165 (99 379-318 715)	158 841 (95 011-312 860)
Government	122 368 (92 235-164 507)	130 158 (95 293-167 175)	128 150 (85 107-166 083)
Physicians (n = 99 077)			
Employed	179 350 (92 767-302 850)	186 147 (101 419-354 267)	200 253 (104 287-363 914)
Self-employed	223 805 (135 639-376 413)	238 274 (119 411-383 309)	249 767 (111 058-365 863)
Government	146 309 (73 154-203 459)	153 334 (78 477-214 940)	164 017 (87 134-264 478)
Pharmacists (n = 26 143)			
Employed	106 407 (85 125-123 689)	115 000 (93 140-131 429)	115 145 (92 260-130 359)
Self-employed	115 974 (75 963-196 612)	115 000 (78 857-209 216)	110 139 (71 885-184 520)
Government	98 952 (79 805-115 294)	109 863 (95 529-124 776)	111 058 (93 118-125 144)
Optometrists (n = 4238)			
Employed	101 730 (742 14-130 539)	100 899 (74 476-128 926)	102 511 (77 097-133 487)
Self-employed	122 452 (77 018-186 091)	106 004 (67 266-153 334)	102 201 (67 645-160 202)
Government	90 072 (67 820-129 874)	102 917 (88 366-127 451)	104 131 (80 770-126 203)
Podiatrists (n = 1164)			
Employed	109 190 (61 845-155 849)	111 265 (81 199-176 731)	128 956 (80 770-205 022)
Self-employed	132 348 (74 214-212 813)	121 831 (71 647-234 382)	110 885 (65 424-209 802)
Government	133 585 (116 180-154 289)	119 411 (109 524-136 774)	121 155 (48 281-146 002)
Chiropractors (n = 6076)			
Employed	65 412 (46 994-94 320)	67 266 (43 810-100 899)	63 080 (43 414-102 511)
Self-employed	86 583 (52 216-177 688)	75 550 (43 810-124 277)	66 632 (38 954-108 458)
Government	NA^b^	79 405 (25 618-87 619)	100 126 (45 433-164 017)
Physical therapists (n = 19 008)			
Employed	67 820 (49 476-81 635)	71 017 (52 692-87 173)	71 758 (53 306-87 110)
Self-employed	82 872 (49 476-140 982)	78 477 (49 689-116 231)	76 883 (49 038-114 715)
Government	65 269 (52 073-80 398)	69 000 (49 488-82 961)	71 758 (56 539-86 109)

Abbreviations: IQR, interquartile range; NA, not available.

^a^ All dollar values are normalized to 2015 dollars using the Consumer Price Index.

^b^ No survey results were available for this category.

**Figure.**
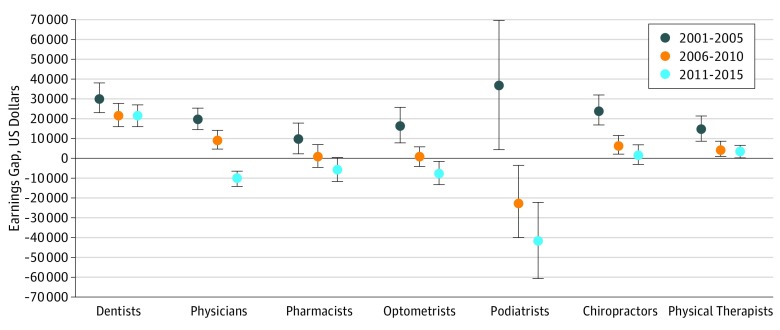
Adjusted Gap in Real Annual Earnings Between Self-employed and Employed Health Care Professionals Annual earnings gap defined as earnings of self-employed health care professionals minus earnings of health care professionals employed by private for-profit or nonprofit organizations. Earnings gap measured in constant 2015 dollars. Estimates come from median regression of log earnings on employment type. Regression includes age, age squared, sex, race, state of residence, and year. Error bars indicate 95% confidence intervals.

## Discussion

Since 2001, the percentage of self-employed health care professionals declined and the gap in annual earnings between self-employed and employed health care professionals narrowed. Employed health care professionals are more likely to be part of large provider groups. Large provider groups may be better able to deal with the increasing complexity of today’s health care economy and therefore better able to pass down this advantage in the form of increased wages to health care professionals.

### Limitations

There are study limitations. Information on physician specialty is not included in the ACS. There may be wide variability among specialties in physician income. If changes over time in employment modality vary by specialty, comparisons could be biased. Because the ACS top-codes income, trends in earnings among high earners could be masked.

## Conclusions

Since 2001, there has been a decline in the percentage of health care professionals identifying themselves as self-employed. Future research is warranted to determine the driving forces behind the shift away from self-employment and the shrinking earnings gap between employed and self-employed health care professionals.
